# Real-world experience of how chlorhexidine bathing affects the acquisition and incidence of vancomycin-resistant enterococci (VRE) in a medical intensive care unit with VRE endemicity: a prospective interrupted time-series study

**DOI:** 10.1186/s13756-021-01030-6

**Published:** 2021-11-10

**Authors:** Jin Woong Suh, Nam Hee Kim, Min Jung Lee, Seoung Eun Lee, Byung Chul Chun, Chang Kyu Lee, Juneyoung Lee, Jong Hun Kim, Sun Bean Kim, Young Kyung Yoon, Jang Wook Sohn, Min Ja Kim

**Affiliations:** 1 Division of Infectious Diseases, Department of Internal Medicine, Korea University Anam Hospital, 73 Goryeodae-ro, Seongbuk-gu, Seoul, 02841 Republic of Korea; 2 Infection Control Unit, Korea University Anam Hospital, Seoul, Republic of Korea; 3 Department of Preventive Medicine, Korea University College of Medicine, Seoul, Republic of Korea; 4 Department of Laboratory Medicine, Korea University College of Medicine, Seoul, Republic of Korea; 5 Department of Medical Statistics, Korea University College of Medicine, Seoul, Republic of Korea; 6 Division of Infectious Diseases, Department of Internal Medicine, Korea University College of Medicine, Seoul, Republic of Korea; 7 Institute of Emerging Infectious Diseases, Korea University, Seoul, Republic of Korea; 8 Present Address: Division of Infectious Diseases, Department of Internal Medicine, CHA Bundang Medical Center, CHA University, Seongnam, Republic of Korea

**Keywords:** Chlorhexidine gluconate, Baths, Vancomycin-resistant enterococci, Acquisition, Intensive care unit, Interrupted time-series analysis

## Abstract

**Background:**

Critically ill patients in intensive care units (ICUs) often acquire opportunistic infections or are colonized by vancomycin-resistant enterococci (VRE), which limits therapeutic options and results in high case-fatality rates. In clinical practice, the beneficial effects of universal chlorhexidine gluconate (CHG) bathing on the control of VRE remain unclear. This study aimed to investigate whether 2% CHG daily bathing reduced the acquisition of VRE in the setting of a medical ICU (MICU) with VRE endemicity.

**Methods:**

This quasi-experimental intervention study was conducted in a 23-bed MICU of a tertiary care hospital in Korea from September 2016 to December 2017. In a prospective, interrupted time-series analysis (ITS) with a 6-month CHG bathing intervention, we compared the acquisition and incidence of VRE and the incidence of methicillin-resistant *Staphylococcus aureus* (MRSA) and carbapenem-resistant *Acinetobacter baumannii* (CRAB) between the pre-intervention and intervention periods. The primary and secondary outcomes were a change in the acquisition of VRE and incidence of VRE, MRSA, or CRAB between the two periods, respectively.

**Results:**

All the adult patients admitted to the MICU were enrolled in the pre-intervention (n = 259) and intervention (n = 242). The overall CHG daily bathing compliance rate was 72.5%. In the ITS, there was a significant intervention effect with a 58% decrease in VRE acquisition (95% CI 7.1–82.1%,* p* = 0.038) following the intervention. However, there was no significant intervention effects on the incidence trend of VRE, MRSA, and CRAB determined by clinical culture between the pre-intervention and intervention periods.

**Conclusion:**

In this real-world study, we concluded that daily bathing with CHG may be an effective measure to reduce VRE cross-transmission among patients in MICU with a high VRE endemicity.

**Supplementary Information:**

The online version contains supplementary material available at 10.1186/s13756-021-01030-6.

## Background

In this era of antimicrobial resistance, patients in intensive care units (ICUs) who are critically ill often contract opportunistic infections or are colonized by multidrug-resistant organisms (MDROs) such as vancomycin-resistant enterococci (VRE), methicillin-resistant *Staphylococcus aureus* (MRSA), and carbapenem-resistant *Acinetobacter baumannii* (CRAB) [1–3]. The MDRO burden in ICUs is also affected by highly antimicrobial-resistant pathogens, for which there are extremely limited treatment options, and which lead to high morbidity and mortality rates [4, 5]. VRE are often endemic in ICUs, where VRE colonization or acquisition is associated with VRE-related infections with significant mortality and morbidity [6, 7]. The persistence of VRE in ICUs is determined through the selective pressure of antibiotic usage, colonization pressure, and patients vulnerable to colonization or infection. Skin contamination can increase the risk of infection through indwelling medical devices or wounds and contribute to cross-transmission of VRE via environmental shedding and the contaminated hands of healthcare workers (HCWs) [8, 9].

In South Korea, infectious diseases due to six types of MDROs have been legally designated for sentinel surveillance since December 2010 [10]. According to the annual report of the Korean National Healthcare-associated Infection Surveillance (KONIS) at 301 ICUs in 216 participating hospitals from July 2018 through June 2019, a total of 4672 major pathogens were isolated from patients with healthcare-associated infections. Of these, 785 (16.8%) isolates were *Enterococcus faecium*, with a vancomycin resistance rate of 55.9% [11]. Our institution has been further challenged by the higher incidence of VRE in ICUs along with an influx of patients from outside hospitals, despite the routine application of evidence-based control measures, such as antibiotic stewardship programs, hand hygiene monitoring, active surveillance cultures (ASC) for rectal VRE, cohort isolation, and environmental surface cleaning [12, 13]. Therefore, we needed an additional, more stringent approach to reduce the cross-transmission of VRE in our ICUs.

Preventing the spread of VRE colonization or infection remains an ongoing challenge. Regimens and efficacy concerning decolonization protocols for patients with VRE have not yet been established [9]. Nevertheless, recent studies have demonstrated that decolonization through the use of chlorhexidine gluconate (CHG) bathing for patients in ICU can prevent hospital-acquired infections (HAIs) and reduce the acquisition rate of MDROs including VRE, MRSA, and multidrug resistant-gram negative bacteria (MDR-GNB) [14–16]. Therefore, further research is needed to evaluate the effects of routine CHG bathing on MDROs in a real-world setting. In this study, we developed a daily bathing protocol using 2% CHG and performed an intervention study in the medical ICU (MICU) to investigate changes in the acquisition of VRE and the incidence of VRE, MRSA, and CRAB using a prospective interrupted time-series (ITS) analysis.

## Methods

### Study design and subjects

During a prospective sequential period, ITS analysis was conducted to evaluate the effects of daily CHG bathing in a 23-bed MICU at Korea University Anam Hospital, a 1048-bed tertiary care teaching hospital in South Korea, from September 2016 to December 2017. We employed a pre-intervention and intervention, quasi-experimental design with equivalent assessment timing to evaluate the effect of CHG bathing implementation after completion of the intervention. We checked the achieved effects and assessed the unexpected effects of the intervention. This enabled detection of changes in real-world data that might be attributable to the intervention.

**Fig. 1:**
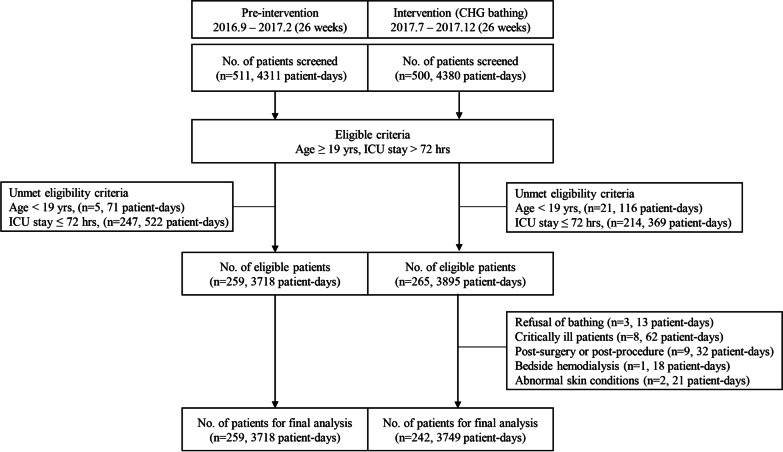
Flowchart of the patients included during the pre-intervention and intervention of the study period. CHG: chlorhexidine gluconate; ICU: intensive care unit

The study comprised two periods: the pre-intervention period from September 2016 to February 2017 (26 weeks) and intervention period from July 2017 to December 2017 (26 weeks). A 6-month intervention with 2% CHG daily bathing was performed, whereas standard bed baths using soap and water were provided twice a week during the pre-intervention periods. An intervening period between two periods was excluded from the analysis, which corresponded to the preparation of the customized CHG bathing protocol and pilot trial (9 weeks), and the following wash-out period (8 weeks). The subjects included patients aged ≥ 19 years, who had been admitted to the MICU for > 72 h (Fig. 1). The primary outcome was a change in the acquisition rate of rectal VRE, as determined using active surveillance culture (ASC). The secondary outcome included a change in the incidence of VRE, MRSA, or CRAB, as determined using clinical cultures.

### Customized 2% chlorhexidine daily bathing protocol

Infection control unit staff nurses developed the CHG bathing protocol based on a previously published CHG decolonization protocol [17]. Commercial CHG cloth-compatible products were not available in South Korea; therefore, cotton wipes impregnated with 2% CHG were prepared daily, using 5% CHG solution (Green Pharmaceutical Co., Ltd, Seoul, South Korea) and warm, sterile distilled water prior to use. Six clean CHG cloth wipes were used to bathe the whole body below the jawline, concentrating on each area of six body sites sequentially (neck, arms, groin/perineum, right leg/foot, left leg/foot, and back of neck, back, and buttocks). Partial bathing was performed for patients who had medical conditions in which they were unable to move easily or moved with pain. CHG bathing was performed once daily by two trained ICU assistants during the entire ICU stay. All the patients were tested for skin contact irritation or allergic reactions to CHG exposure prior to bathing. Patients with MDRO isolation were bathed last. Patients in critical condition, dermatitis, or abnormal skin conditions were also excluded from CHG daily bathing, depending on the patient’s condition.

CHG bathing compliance was assessed daily, based on documentation provided by an infection control practitioner. A patient receiving full or partial bathing was considered “compliant”. Compliance rate was calculated through dividing the total number of the “compliant” patient-days by the total number of patient-days.

Skin swab cultures of the body sites at high risk of MDRO acquisition were monitored in a small number of randomly selected patients prior to the pilot trial (n = 4) and during the 6-month intervention (two patients per month, n = 12), including patients who had a longer ICU stay of > 30 days and MDRO isolates from the clinical culture (Additional file 1: Table S1).

### Routine infection control and prevention program in the ICU

The hospital runs a routine ICU infection control and prevention program, including ASC on ICU admission for rectal VRE and nasal MRSA, a follow-up weekly surveillance rectal culture for VRE acquisition, single room or cohort isolation of patients with VRE, monitoring of hand hygiene adherence for HCWs, monitoring of HAIs, and the electronic antibiotic stewardship program. Patients with a previous MDRO colonization were placed on contact precautions and cohort isolation on admission to the ICU. The infection control staff monitored all the measures on a monthly basis.

### Data collection

Demographic and clinical data concerning patients in the ICU were collected through reviewing hospital electronic medical records. We collected data on age, sex, admission department, length of ICU and hospital stay, Acute Physiology and Chronic Health Evaluation (APACHE) II score, medical devices used (mechanical ventilator, urinary catheter, and central venous catheter), and recent surgery and clinical outcomes.

Microbiological data were collected from hospital electronic medical records. Data on MDRO surveillance and ICU-acquired device-associated infections (DAIs) were also obtained from the infection control unit database. The acquisition of rectal VRE using ASC and incidence of VRE, MRSA, and CRAB in clinical cultures were collected weekly for ITS analysis.

### Definitions

The compliance rate with the CHG bathing intervention was calculated as the total number of CHG bathing days divided by the total number of patient-days as denominator and expressed in percentage. Since the patients with a stay ≤ 72 h in ICUs were excluded, the patient-days for these patients were subtracted from the denominator. VRE acquisition was evaluated for all the patients who had no previous VRE isolation, a negative ASC result for VRE on ICU admission, and a positive follow-up weekly ASC result for VRE. The incidence of VRE, MRSA, and CRAB was defined as newly acquired positive clinical cultures obtained > 48 h after ICU admission. Clinical cultures were obtained depending on a patient’s clinical status, as determined by the medical staff. Only the first isolate from a single body site per patient was included in the analysis. Identification and susceptibility determination of MDROs were undertaken in the hospital microbiological laboratory. The laboratory definition of each MDRO was determined according to the Clinical and Laboratory Standards Institute criteria [18].

The definitions of MICU-acquired DAIs, including central line-associated bloodstream infection (CLA-BSI), catheter-associated urinary tract infection (CA-UTI), and ventilator-associated pneumonia (VAP), were used based on those of the KONIS national surveillance program, as our hospital has been participating in the ICU module of the program since 2006 [19–21].

### Statistical analyses

We compared continuous variables, expressed as median and inter-quartile range (IQR), using Student’s t- or the Mann–Whitney U tests, depending on their distribution. Categorical variables were analyzed using χ^2^- or Fisher’s exact tests. The VRE acquisition rate and the incidence rates of MDROs were calculated as the total number of episodes divided by the total number of patient-days as denominator and expressed per 1000 patient-days. DAI rates were calculated as the total number of cases per 1000 device-days.

ITS was used to statistically measure the changes in the level and slope of the trends over time for the acquisition of rectal VRE and incidence of MDROs between the pre-intervention and intervention periods. We calculated the final multivariable model with a significant parameter (the use of mechanical ventilator) using stepwise backward variable selection. The outcome and offset parameters were the weekly number of cases and the logarithmized patient days, respectively. Overdispersion was tested for the distribution of cases, including potential confounding parameter such as the use of mechanical ventilators. Since there was no overdispersion, we calculated the change in the level and slope of the series using a segmented Poisson regression model. The model used for the analysis is as follows:

Log[E(Y∣X1,X2,X3,X4)] = *β*_*0*_ + *β*_*1*_log(X1) + *β*_*2*_log(X2) + *β*_*3*_log(X3) + *β*_*4*_log(X4) + *e*_*t*_

(X1: time, X2: intervention, X3: time after intervention, and X4: covariates), where log[E(Y∣X1,X2,X3,X4)] was the VRE acquisition and MDRO incidence per patient-days, time was the number of weeks starting in September 2016, *β*_0_ estimated the intercept at the beginning of the time series, *β*_1_ estimated the log-linear trend of the pre-intervention period, where X1 was a continuous variable indicating the time in weeks at time t from the initiation of the study period, *β*_2_ estimated the level changes in incidence, where intervention_t_ = 0 was pre-intervention, and intervention_t_ = 1 was intervention, *β*_3_ estimated the change in weekly trend from pre-intervention to intervention, *β*_*4*_ estimated the effects of significant covariates, and *e*_t_ was the random error at time *t*.

Incidence rate ratio (IRR) and the percent change as two standardized effect sizes were obtained as a ratio of change in level and trend between the pre-intervention and intervention periods [22]. A change in level represented how the outcome level had changed from the last observation prior to the intervention to the first one after, based on the Poisson model predictions and not on a difference between observed versus predicted levels. Moreover, a change in level represented a direct change, whereas a change in slope represented a sustained effect (if slope goes down) or unsustainable effect (if slope goes up, after level change down) [23]. A negative change in the level and slope indicated a reduction in the infection rates. A *p*-value of < 0.05 was interpreted as a significant association with intervention efficacy. ITS analysis was performed using package R version 3.6.1 (R Foundation for Statistical Computing, Vienna, Austria) software.

## Results

### Compliance to 2% CHG bathing

A total of 501 patients were enrolled (pre-intervention, n = 259; intervention, n = 242). The overall compliance with the 2% CHG daily bathing was 72.5%, including full (66.6%) and partial bathing (5.9%). The most common body site excluded from the partial CHG bathing was the back, followed by the upper trunk and extremities in decreasing order of frequency. No patient had an allergic reaction to chlorhexidine. The reasons for non-compliance to daily bathing were identified: the barriers to CHG bathing in the MICU setting included patient in critical conditions, the presence of dermatitis and/or abnormal skin conditions, patient’s refusal to bathe, and the absence during the bathing time in decreasing frequency (Table 1).

**Table 1:** Compliance with 2% CHG bathing and reasons for failure to perform CHG bathing in 242 patients in MICU during the intervention period

CHG daily bathing	Patient-days (%)
Subjects (n = 242)	3749
Compliance with CHG bathing (%)	
Full bathing	2498 (66.6)
Partial bathing^a^	221 (5.9)
Subtotal	2719 (72.5)
Reasons for non-compliance (%)	
Critically ill^b^	310 (8.2)
Dermatitis/abnormal skin conditions	195 (5.2)
Patient’s refusal to bathe	190 (5.1)
Absence^c^	142 (3.8)
Mechanical barrier^d^	104 (2.8)
Post-surgery or post-procedures	52 (1.4)
Bedside hemodialysis	37 (1.0)
Subtotal	1030 (27.5)

CHG, chlorhexidine gluconate; MICU, medical intensive care unit

^a^Full bathing except the back of the body, upper trunk, or extremity

^b^Patients with unstable vital signs due to serious illness, receiving continuous renal replacement therapy, or dying patients

^c^Absence due to conventional hemodialysis, surgery, or other procedures

^d^Patient with physical restraint or surgical drainage

### HCW hand hygiene adherence

HCW hand hygiene adherence in the ICU was routinely monitored using the World Health Organization’s Five Moments for Hand Hygiene protocol in the daytime by a validated and trained observer and adherence was reported on a monthly basis  [24]. The total number of hand hygiene opportunities for HCWs observed in the MICU in pre-intervention and intervention periods were 3165 and 2193, respectively. There was no significant difference in the overall hand hygiene adherence rates among the registered nurses, nurse aids, and medical doctors between the two periods (registered nurses, 67.97 ± 4.72% vs. 70.41 ± 5.44%, *p* = 0.425; nurse aids, 80.4 ± 8.03% vs. 86.32 ± 3.04%, *p* = 0.139; medical doctors, 63.52 ± 3.71% vs. 71 ± 9.06%, *p* = 0.091: Additional file 1: Table S2).

### Baseline characteristics

The baseline characteristics of the patients in MICU were compared between the pre-intervention and intervention periods (Table 2). No significant differences were observed in terms of sex, age, comorbidities, and a receipt of surgery between the two periods. The patients in intervention presented higher APACHE II scores than those in pre-intervention [18 (IQR 13 − 23) vs. 24 (IQR 16–32), *p* < 0.001]. The overall in-hospital mortality was 34.9% during the study period, which did not differ significantly between the two periods. The median length of MICU stay in pre-intervention and intervention was 13 days each (IQR 11–15, *p* = 0.723). The rectal VRE carriage rates among the patients at the time of admission to the MICU, determined using ASC were 6.6% (17/259) in pre-intervention and 4.5% (11/242) in intervention (*p* = 0.326). The percentages of patients who received at least one follow-up ASC in pre-intervention and intervention were 82.2% (213/259) and 81.4% (197/242), respectively (*p* = 0.809). The device utilization ratio for urinary catheter and central line in pre-intervention was higher than that in intervention period, although the difference was not significant. The ventilator utilization ratio was significantly higher in intervention than in pre-intervention (*p* < 0.001). There was no significant difference in the incidence of CLA-BSI and CA-UTI between the two periods, and no patients with VAP were reported in either period of the study.

**Table 2:** Baseline demographics, rates of the device-associated infections, and acquisition and incidence of MDROs in patients in the MICU during the study period

	MICU		
	Pre-intervention (26 weeks)	Intervention (26 weeks)	*p*-value
No. of patients	259	242	
Age, years, median (IQR),	74 (62–81)	73 (57–81)	0.146^d^
Male, n (%)	155 (59.8)	140 (57.9)	0.650^e^
APACHE II score, median (IQR)	18 (13–23)	24 (16–32)	< 0.001^d^
VRE rectal carriage on ICU admission, n (%)	17 (6.6)	11 (4.5)	0.326^e^
Receipt of surgery, n (%)	95 (36.7)	81 (33.5)	0.452^e^
Length of MICU stay, days, median (IQR)	13 (11–15)	13 (11–15)	0.723^d^
Length of hospital stay, days, median (IQR)	25 (17–49)	24 (14–45)	0.699^d^
In-hospital mortality, n (%)	98 (37.8)	77 (31.8)	0.158^e^
Device utilization ratio^a^			
Mechanical ventilator	0.50	0.63	< 0.001^e^
Urinary catheter	0.97	0.93	0.226^e^
Central venous catheter	0.64	0.63	0.927^e^
Device-related infections^b^			
CLA-BSI	1.25	1.28	0.983^e^
CA-UTI, n (%)	1.95	2.40	0.781^e^
VRE acquisition rate by weekly active surveillance culture^c^			
Rectal VRE	20.17	9.34	< 0.001^e^
MDRO incidence rate by clinical cultures^c^			
VRE	5.11	1.60	0.006^e^
MRSA	5.11	7.74	0.104^e^
CRAB	11.02	11.20	0.850^e^

CA-UTI, catheter-associated urinary tract infection; CLA-BSI, central line associated blood stream infection; ICU, intensive care unit; IQR, interquartile range; MDROs, multidrug-resistant organisms; MICU, medical intensive care unit; CRAB, carbapenem-resistant *A. baumannii;* MRSA, methicillin-resistant *S. aureus*; VAP, ventilator-associated pneumonia; VRE, vancomycin-resistant enterococci

^a^Device utilization ratio is calculated by dividing the number of device-days by the number of patient-days, unless otherwise stated

^b^The data are expressed as the total number of cases of device-associated infections per 1000 device-days, unless otherwise stated

^c^The data are expressed as the number of isolates per 1000 patient-days, unless otherwise stated

^d^Student’s t-test

^e^Chi-square test

### Interrupted time-series analysis

#### Outcomes

**Fig. 2:**
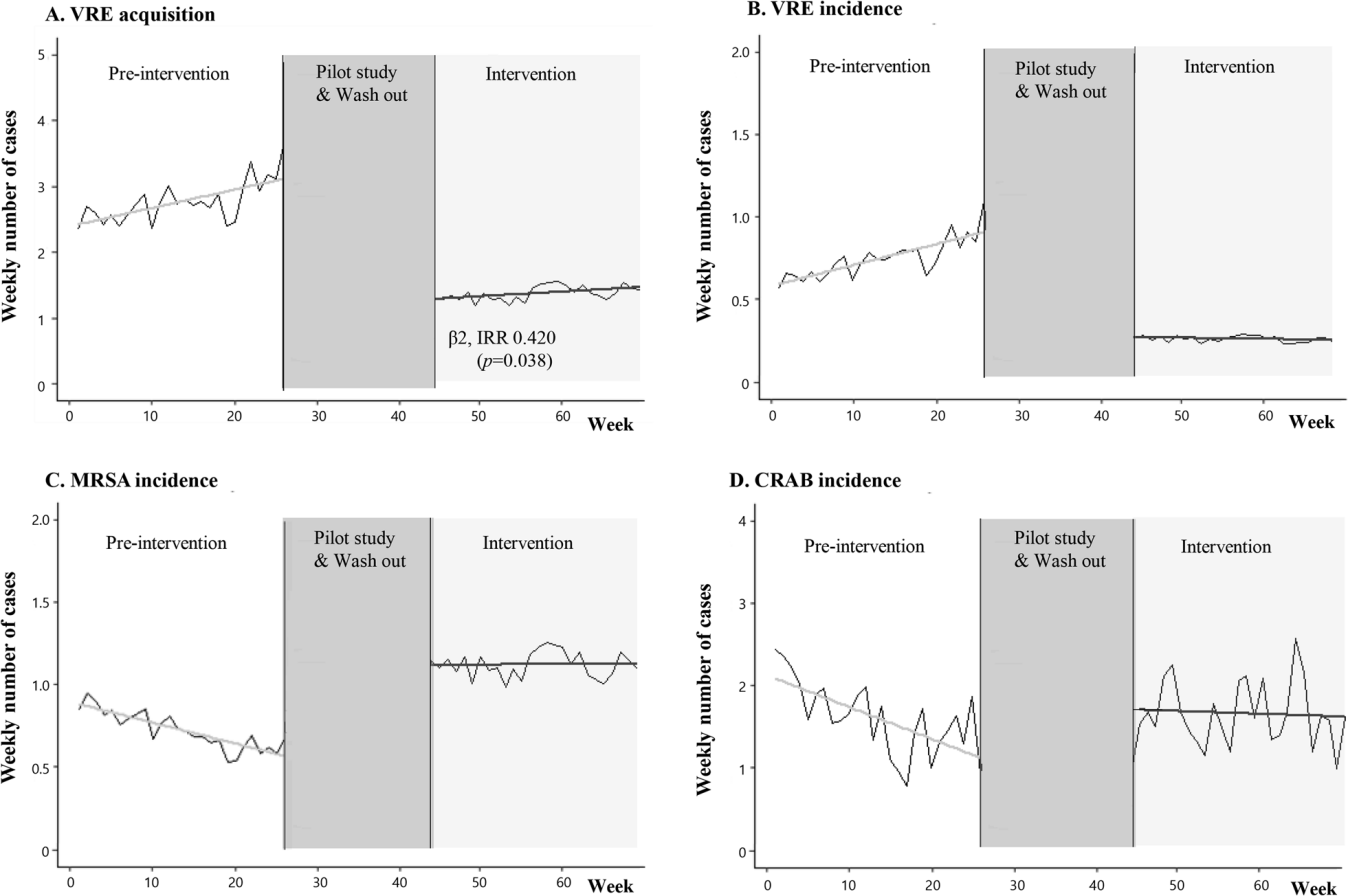
Interrupted time-series analysis of VRE acquisition and MDROs incidence in MICU patients after daily CHG bathing. **A** VRE acquisition, **B** VRE incidence, **C** MRSA incidence, and **D** CRAB incidence. CRAB: carbapenem-resistant *Acinetobacter baumannii*; MICU: medical intensive care unit; MRSA: methicillin-resistant *Staphylococcus aureus*; VRE: vancomycin-resistant enterococci

The overall rectal VRE acquisition rate decreased significantly between pre-intervention and intervention periods (20.17 vs. 9.34 per 1000 patient-days, *p* < 0.001: Table 2). The overall incidence of MRSA and CRAB per 1000 patient-days in clinical cultures were not significantly different between the two periods. The ITS analysis revealed the IRR and percent change calculated from the IRR for the model effects (Table 3). In the VRE acquisition, there was a significant intervention effect (*β*_*2*_*)* with a 58% decrease (95% CI 7.1–82.1%) following the intervention (*p* = 0.038). However, there was no significant difference in the trend (*β*_*3*_) of VRE acquisition between the pre-intervention and intervention periods. There was no significant difference in the intervention effect (*β*_*2*_) and trend (*β*_*3*_) on the incidence of VRE, MRSA, and CRAB by clinical cultures between the two periods (Fig. 2).

**Table 3:** Incidence Rate Ratio and associated percent changes for an interrupted time series analysis model of the VRE acquisition and incidence of MDROs in the MICU during the study periods

	Final multivariable model^a^			
	Regression coefficient (95% CI)	*p*-value^b^	IRR (95% CI)	Percent change^c^ (95% CI)
Rectal VRE acquisition detected according to weekly surveillance				
Intercept (β_0_)	− 4.029 (− 4.655 to − 3.436)	< 0.001	0.018 (0.010 to 0.032)	− 98.2 (− 99 to − 96.8)
Pre-intervention slope (β_1_)	0.008 (− 0.022 to 0.038)	0.065	1.008 (0.978 to 1.039)	0.8 (− 2.2 to 3.9)
Intervention level change (β_2_)	− 0.868 (− 1.721 to − 0.074)	0.038	0.420 (0.179 to 0.929)	− 58.0 (− 82.1 to − 7.1)
Intervention slope change (β_3_)	− 0.007 (− 0.061 to 0.048)	0.812	1.004 (0.899 to 1.120)	0.4 (− 10.1 to 12)
MDROs prevalence in clinical cultures				
VRE				
Intercept (β_0_)	− 5.572 (− 6.905 to − 4.379)	< 0.001	0.004 (0.001 to 0.013)	− 99.6 (− 99.9 to − 98.7)
Pre-intervention slope (β_1_)	0.015 (− 0.046 to 0.076)	0.629	1.015 (0.955 to 1.079)	1.5 (− 4.5 to 7.9)
Intervention level change (β_2_)	− 1.248 (− 3.334 to 0.442)	0.183	0.287 (0.036 to 1.556)	− 71.3 (− 96.4 to 55.6)
Intervention slope change (β_3_)	− 0.201 (− 0.148 to 0.106)	0.744	0.979 (0.862 to 1.112)	− 2.1 (− 13.8 to 11.2)
MRSA				
Intercept (β_0_)	− 5.070 (− 6.184 to − 4.084)	< 0.001	0.006 (0.002 to 0.018)	− 99.4 (− 99.8 to − 98.2)
Pre-intervention slope (β_1_)	− 0.019 (− 0.081 to 0.412)	0.542	0.981 (0.924 to 1.043)	− 1.9 (− 7.6 to 4.3)
Intervention level change (β_2_)	0.719 (− 0.064 to 0.096)	0.238	2.053 (0.621 to 6.785)	105.3 (− 6.2 to 578.5)
Intervention slope change (β_3_)	0.015 (− 0.163 to 0.184)	0.704	1.016 (0.938 to 1.100)	1.6 (− 6.2 to 10.0)
CRAB				
Intercept (β_0_)	− 3.741 (− 4.490 to − 3.042)	< 0.001	0.024 (0.012 to 0.049)	− 97.6 (− 98.8 to − 95.1)
Pre-intervention slope (β_1_)	− 0.021 (− 0.061 to 0.019)	0.310	0.980 (0.941 to 1.020)	− 2.0 (− 5.9 to 2.0)
Intervention level change (β_2_)	0.580 (− 0.325 to 1.491)	0.208	1.785 (0.724 to 4.403)	78.5 (− 27.6 to 340.3)
Intervention slope change (β_3_)	0.004 (− 0.054 to 0.064)	0.886	1.004 (0.947 to 1.065)	0.4 (− 5.3 to 6.5)

CI, confidence interval; IRR, incidence rate ratio; CRAB, carbapenem-resistant *Acinetobacter baumannii;* MDRO, multidrug-resistant organism; MICU, medical intensive care unit; MRSA, methicillin-resistant *S. aureus*; VRE, vancomycin-resistant enterococci

^a^Final multivariable model includes the use of mechanical ventilator as confounding parameters

^b^Interrupted time series with segmented Poisson regression model

^c^(IRR − 1) × 100

## Discussion

This quasi-experimental, intervention study investigated whether daily bathing with 2% CHG as an adjunctive intervention could reduce the VRE acquisition and the incidence of VRE, MRSA, or CRAB in a VRE-endemic MICU. In the ITS analysis, we have shown a significant intervention-associated reduction in the acquisition of rectal VRE, following the intervention of 2% CHG daily bathing at a compliance rate of 72.5%. These results suggest that daily CHG bathing could reduce cross-transmission of VRE in patients in the MICU setting with a high VRE endemicity.

Patients with rectal VRE carriage on ICU admission often lead to subsequent colonization of other patients, which may play an important role in the spread and persistence of VRE in the ICU [25]. High VRE endemicity has become stabilized in our ICU settings despite weekly active surveillance for rectal VRE and other infection control measures. However, our hospital has been faced with a constant influx of patients colonized with MDROs from outside healthcare facilities since 2016, when it was designated a regional emergency center [13, 26]. Therefore, the infection control staff developed a daily CHG bathing protocol to overcome the challenges of priority VRE control.

In this study, ITS analysis showed that daily CHG bathing reduced the acquisition rate of rectal VRE, but not the incidence of VRE in clinical cultures between the pre-intervention and intervention periods. Our results are consistent with those of two previous studies on the effect of CHG bathing in the ICU settings [27, 28]. A quasi-experimental, multi-center trial performed in six ICUs reported a significant decrease of 45% in the acquisition of VRE by the end of the intervention [27]. Another prospective clinical trial at a single center reported a significant decrease the incidence of VRE acquisition decreased from 26 colonizations per 1000 patient-days to 9 per 1000 patient-days in the setting of MICU following the intervention [28]. Furthermore, two meta-analyses showed that patients with CHG bathing had a significantly lower risk of VRE colonization [29, 30].

A higher compliance rate with the CHG bathing protocol might have further improved the decrease in colonization of MDROs and the HAI rate. In this study, the compliance rate of 72.5%, including full and partial daily CHG bathing was compatible with the reported compliance rates ranging from 70 to 100% in previous studies [31–33]. We evaluated that CHG daily bathing compliance was affected due to multiple barriers in MICU settings. A trained ICU assistant helping patients with CHG bathing was likely to reduce the likelihood of a patient declining to undertake a CHG bath; however, other multiple barriers to CHG daily bathing mostly related to a patient’s clinical severity, which reduced the compliance rate. In our study, the continuing efforts to monitor and provide feedback to staff nurses with increasing compliance rate of CHG daily bathing reflected a real-world setting in the MICU with heterogeneous population and health outcomes. The findings from our real-world study provide further evidence-based information concerning the advantages and disadvantages of daily CHG bathing, as used in an ICU setting.

At our institute, the overall proportions of MRSA and CRAB in the clinical isolates in the MICU were high throughout the study period, accounting for approximately 81.5% (172/211) of all *Staphylococcus aureus* isolates and 86.3% (233/270) of all *Acinetobacter baumannii* isolates, respectively. However, the ITS analysis failed to show a significant reduction in MRSA and CRAB incidence in clinical cultures after the intervention. There have been two, randomized controlled studies targeting MRSA and/or VRE in ICUs following a CHG daily bathing intervention. Huang et al*.* reported a significant decrease in the prevalence of MRSA clinical isolates following targeted or universal decolonization, including CHG bathing and intranasal mupirocin for 5 days [17]. Another multicenter randomized controlled trial by Climo et al*.* demonstrated a significant decrease in the acquisition of VRE and MRSA in patients in ICU following interventions involving CHG bathing and active surveillance cultures for perirectal VRE and nasal MRSA [27]. These two studies reported a compliance rate of approximately 85%. Unlike these studies, our study protocol focused primarily on VRE control, which included active surveillance cultures on admission and on a weekly basis and single room or cohort isolation in addition to a CHG daily bathing intervention at a compliance rate of 72.5%. On the other hand, our protocol did not specifically include MRSA-targeted strategies, such as active surveillance culture for MRSA nasal carriage and decolonization with intranasal mupirocin. For the effective control of MDROs in ICU settings with CHG daily bathing, we speculate that it is necessary to prepare strategy protocols targeting the specific pathogens, considering the magnitude of the compliance rate and duration of the intervention.

In our study, CHG bathing was not associated with significant reductions in the incidence of DAIs (CLA-BSI and CA-UTI). This might be related to the low number of DAIs during the entire study period. The previous studies have reported inconsistent results of the effect of CHG bathing on the incidence of DAIs in ICUs [34–36]. In a single-center randomized controlled trial performed in 5 ICUs, the intervention of CHG bathing did not reduce the incidence of HAIs (CLA-BSIs, CA-UTIs, VAP, and *Clostridium difficile* infections) in patients [34]. In another study, no significant decrease was reported in the incidence of ICU-acquired CLA-BSI between the control and CHG bathing periods [35]. In contrast, Popovich et al. reported a significant decrease in CLA-BSI following CHG bathing [36]. It is unclear whether the effect of CHG bathing is dependent on the baseline incidence rate of HAIs.

This study has several limitations. First, the study was conducted in a single center; therefore, generalizing the potential effects of CHG bathing to other hospitals’ settings might be difficult. A multicenter study with real-world ICU settings can provide the potential effects of CHG bathing against MDRO control. Second, not all the analyzed patients could receive at least one follow-up weekly VRE surveillance during the study periods. Approximately eighteen percent of the analyzed patients failed to follow-up with VRE surveillance cultures due to ICU stay < 7 days, death, or transfer to general wards. Third, in the setting of MICU of our study, the proportions of patients with ≤ 72 h-ICU stay for pre-intervention and intervention periods were 48.3% (n = 247) and 42.8% (n = 214), respectively. However, the proportions of the patient-days for the two periods were 12.1% (522 patient-days) and 8.4% (369 patient-days), respectively. Fourth, this was a quasi-experimental study, which was susceptible to bias and confounding. We did not consider the randomization and control arm in the study design. Fifth, the pre-intervention and intervention periods did not cover the same months and seasons, which could have played a role in the effect of intervention. Last, our study did not investigate MDROs for development of CHG resistance. Future studies are needed to evaluate MDRO isolates for CHG-resistance.

In this era of antibiotic resistance, MDROs are prevalent worldwide. Multiple intervention strategies are needed for optimal infection control of MDROs, including continuing education, antibiotic stewardship, active surveillance, contact precautions, and environmental control measures [37]. Effective measures for MDRO decolonization remain limited in ICU settings. CHG bathing, as a universal decolonization strategy might be an adjunctive control measure to reduce cross-transmission of VRE until more effective measures become available [38].

## Conclusions

This real-world intervention study showed that daily bathing with 2% CHG at a compliance rate of 72.5% could be an effective adjunctive control measure to reduce the acquisition rate of VRE in the ICU where VRE is endemic.

## Supplementary Information

**Additional file 1:** Detailed information concerning the MDRO distribution from skin swab cultures prior to the pilot study and during the six-month intervention of 2% CHG bathing, the hand hygiene adherence rate for healthcare workers in the MICU during the study period, and the isolation of microorganisms from patients with DAIs in the MICU during the study period. **Table S1.** Skin swab cultures for MDROs from the representative body sites of patients in MICU prior to the pilot trial and during the six-month intervention of 2% CHG daily bathing. **Table S2.** Hand hygiene adherence rate for HCWs in the MICU observed at the World Health Organization’s Five Moments of Hand Hygiene during the study period. **Table S3.** Isolation of microorganisms from the patients with device-associated hospital-acquired infections in the MICU during the study period.

## Data Availability

The original contributions presented in the study are included in the article/Additional file 1; further inquiries can be directed to the corresponding author.

## Abbreviations

ASC: Active surveillance cultures
CA-UTI: Catheter-associated urinary tract infection
CHG: Chlorhexidine gluconate
CI: Confidence interval
CLA-BSI: Central line-associated blood stream infection
CoN-staphylococci: Coagulase-negative staphylococci
CRAB: Carbapenem-resistant *Acinetobacter baumannii*
DAIs: Device-associated infections
HAIs: Hospital-acquired infections
HCWs: Healthcare workers
IQR: Interquartile range
ITS: Interrupted time-series
KONIS: Korean national healthcare-associated infections surveillance system
MDR-GNB: Multidrug resistant-gram negative bacteria
MDROs: Multidrug-resistant organisms
MICU: Medical intensive care unit
MRSA: Methicillin-resistant *Staphylococcus aureus*
RC: Regression coefficient
SD: Standard deviation
VAP: Ventilator-associated pneumonia
VRE: Vancomycin-resistant enterococci
IRR: Incidence rate ratio
