# Supplementary material for: Real-world experience of how chlorhexidine bathing affects the acquisition and incidence of vancomycin-resistant enterococci (VRE) in a medical intensive care unit with VRE endemicity: a prospective interrupted time-series study
Source: Antimicrob Resist Infect Control. 2021 Nov 10;10:160. doi: 10.1186/s13756-021-01030-6 (PMC8579179; doi:10.1186/s13756-021-01030-6)
Supplement: Supplementary file 1 — Additional file 1: Detailed information concerning the MDRO distribution from skin swab cultures prior to the pilot study and during the six-month intervention of 2% CHG bathing, the hand hygiene adherence rate for healthcare workers in the MICU during the study period, and the isolation of microorganisms from patients with DAIs in the MICU during the study period. Table S1. Skin swab cultures for MDROs from the representative body sites of patients in MICU prior to the pilot trial and during the six-month intervention of 2% CHG daily bathing. Table S2. Hand hygiene adherence rate for HCWs in the MICU observed at the World Health Organization’s Five Moments of Hand Hygiene during the study period. Table S3. Isolation of microorganisms from the patients with device-associated hospital-acquired infections in the MICU during the study period. [file 13756_2021_1030_MOESM1_ESM.docx]

**Supplementary materials**

Table S1. Skin swab cultures for MDROs from the representative body sites of patients in MICU before prior to the pilot trial and during the six-month intervention of 2% CHG daily bathing.

|  | No. of patients with skin cultures ^a^ (positivity/ tested) according to the body site | | | | | |
| --- | --- | --- | --- | --- | --- | --- |
| MDRO  (No. of isolates) | Anterior neck | Axilla ^b^ | Groin ^b^ | Popliteal fossa ^b^ | Posterior neck | Buttock |
| Before Prior to the pilot trial (four patients subjected, n = 4) | | | | | | |
| MRSA (0) | 0/4 | 0/4 | 0/4 | 0/4 | 0/4 | 0/4 |
| VRE (3) | 0/4 | 0/4 | 2/4 | 1/4 | 0/4 | 0/4 |
| CRAB (3) | 1/4 | 1/4 | 0/4 | 0/4 | 1/4 | 0/4 |
| During the six-month intervention (two patients subjected each month, n = 12) | | | | | | |
| MRSA (4) | 1/12 | 1/12 | 0/12 | 0/12 | 2/12 | 0/12 |
| VRE (4) | 1/12 | 0/12 | 0/12 | 0/12 | 0/12 | 3/12 |
| CRAB (9) | 0/12 | 0/12 | 0/12 | 0/12 | 3/12 | 6/12 |
| CRPA (1) | 0/12 | 0/12 | 0/12 | 0/12 | 0/12 | 1/12 |
| CRE (1) | 0/12 | 0/12 | 0/12 | 0/12 | 0/12 | 1/12 |

^a^ Swab culture was performed in the area of a 5-cm square for each body region.

^b^ Swab culture was performed on both sides.

Abbreviations: CRAB, carbapenem-resistant *Acinetobacter baumannii*; CRE, carbapenem-resistant Enterobacteriaceae; CRPA, carbapenem-resistant *Pseudomonas aeruginosa*; MDROs, multidrug-resistant organisms; MRSA, Methicillin-resistant *Staphylococcus aureus*; VRE, vancomycin-resistant enterococci

Table S2. Hand hygiene adherence rate for HCWs in the MICU observed at the World Health Organization’s Five Moments of Hand Hygiene during the study period.

|  |  | Registered nurse | | Nurse aids | | Medical doctor | |
| --- | --- | --- | --- | --- | --- | --- | --- |
| Year | Month | Total no. of opportunities  observed | Adherence rate (%) | Total no. of opportunities  observed | Adherence rate (%) | Total no. of opportunities  observed | Adherence rate (%) |
| Pre-intervention | | | | | | | |
| 2016 | SEP | 339 | 67.0 | 296 | 74.30 | 37 | 59.45 |
|  | OCT | 235 | 70.0 | 232 | 75.0 | 20 | 65.0 |
|  | NOV | 367 | 70.03 | 336 | 75.0 | 28 | 64.29 |
|  | DEC | 478 | 71.34 | 240 | 86.30 | 81 | 69.10 |
| 2017 | JAN | 153 | 58.80 | 36 | 77.78 | 27 | 59.26 |
|  | FEB | 160 | 70.62 | 50 | 94.0 | 50 | 64.0 |
| Subtotal^a^ | | 1732 | 67.97 ± 4.72 | 1190 | 80.40 ± 8.03 | 243 | 63.52 ± 3.71 |
| Intervention | | | | | | | |
| 2017 | JUL | 200 | 61.0 | 75 | 88.0 | 40 | 62.50 |
|  | AUG | 210 | 72.85 | 86 | 87.20 | 81 | 65.40 |
|  | SEP | 200 | 71.0 | 100 | 81.0 | 52 | 65.40 |
|  | OCT | 240 | 70.83 | 80 | 86.25 | 47 | 70.20 |
|  | NOV | 250 | 69.20 | 110 | 85.45 | 49 | 75.50 |
|  | DEC | 250 | 77.60 | 100 | 90.0 | 23 | 86.95 |
| Subtotal^a^ | | 1350 | 70.41 ± 5.44 | 551 | 86.32 ± 3.04 | 292 | 71.00 ± 9.06 |
| *p*-value | |  | 0.425 |  | 0.139 |  | 0.091 |

^a^Adherence rate data are expressed as mean ± standard deviation (SD) unless otherwise stated.

Abbreviations: HCWs, healthcare workers; MICU, medical intensive care unit

Table S3. Isolation of microorganisms from the patients with device-associated hospital-acquired infections in the MICU during the study period

|  | Pre-intervention | Intervention |  | Pre-intervention | Intervention |
| --- | --- | --- | --- | --- | --- |
| CLA-BSI, n |  |  | CA-UTI, n |  |  |
| *K. pneumoniae* | 1 | 1 | *E.* *coli* | 3 | 3 |
| *S. epidermidis* | 1 |  | *P.* *aeruginosa* |  | 3 |
| *S. aureus* | 1 |  | *K.* *oxytoca* | 1 | 1 |
| *A. baumanii* |  | 1 | *C.* *freundii* |  | 1 |
| *E. faecalis* |  | 1 | CoN-staphylococci | 1 |  |
|  |  |  | *E.* *faecium* | 2 | 1 |
| Total | 3 | 3 | Total | 7 | 9 |

Data are expressed as the number of microorganisms isolated unless otherwise stated.

Abbreviations: CAU-TI, catheter-associated urinary tract infection; CLA-BSI, central line-associated bloodstream infection; CoN-staphylococci, coagulase-negative staphylococci
